# Tuberculous brain abscess mimicking stroke in HIV-negative patient

**DOI:** 10.1016/j.bjid.2025.104606

**Published:** 2025-12-23

**Authors:** Emerson dos Santos Hoffmann, Cecilia Freire Lopes, Giulia De Bastiani Graziottin, Maíra Cristina Velho, Mateus Swarovsky Helfer

**Affiliations:** a Moinhos de Vento Hospital, Serviço de Doenças Infecciosas, Porto Alegre, RS, Brazil; b Hospital de Clínicas de Porto Alegre, Serviço de Doenças Infecciosas, Porto Alegre, RS, Brazil; c Hospital de Clínicas de Porto Alegre, Serviço de Neurocirurgia, Porto Alegre, RS, Brazil

A 58-year-old male recycling worker was admitted to the emergency department with sudden-onset left-sided hemiparesis and altered mental status, prompting an initial evaluation for acute stroke. The patient also had a one-year history of progressive cognitive impairment, confusion, behavioral changes, and weight loss, with a BMI of 16.45 kg/m^2^ upon admission. Brain magnetic resonance imaging revealed a large intra-axial expansile lesion centered in the right parietal lobe, predominantly cystic and necrotic in appearance, containing fluid-fluid levels and a diffusion restriction component in its posterior aspect, suggestive of an abscess (Fig. 1).

**Fig. 1:**
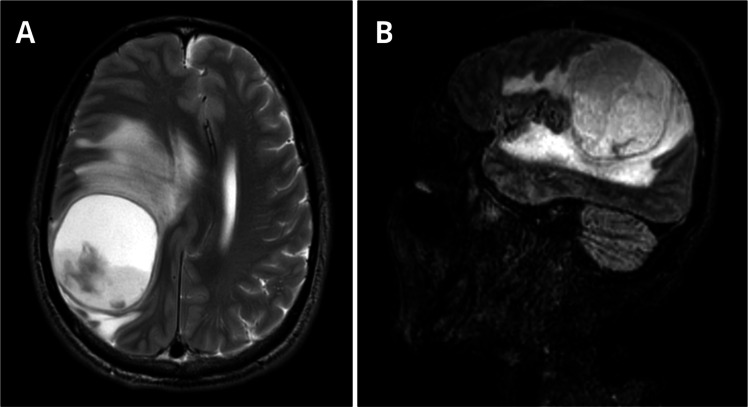
Axial T2-weighted MRI (A) shows a lesion measuring 5.9 × 5.4 × 5.4 cm (CC × LL × AP) and exerts a compressive effect on the brain parenchyma, with hyperintense areas in the white matter secondary to vasogenic edema, accompanied by subfalcine herniation and right uncal herniation into the suprasellar cistern. There is an approximate 1.7 cm midline shift to the left. Peripheral rim enhancement without surrounding capsule irregularity is observed, findings that are suggestive of a tuberculous abscess rather than a pyogenic one. Sagittal MRI on T1 complements the visualization of the lesion (B).

Family members reported that the patient had been receiving treatment for pulmonary Tuberculosis (TB) for three months without adherence. A new chest CT scan was performed, which revealed pulmonary cavities with thickened walls in the apical-posterior segment of the left upper lobe and the superior segment of the left lower lobe. The smear of sputum microscopy was negative for acid-fast bacilli. HIV serology was negative.

The patient underwent abscess resection, revealed an encapsulated mass containing purulent material. Polymerase chain reaction analysis (GeneXpert) of the purulent fluid confirmed the presence of *Mycobacterium tuberculosis* with no detected resistance to rifampin (Fig. 2).

**Fig. 2:**
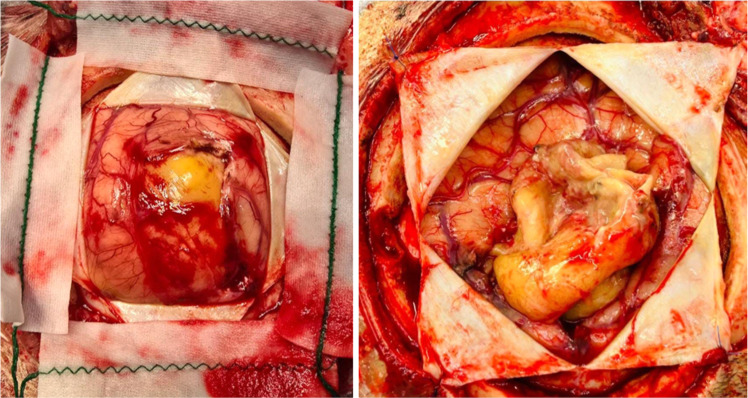
Photo from the surgery showing an encapsulated lesion, well-demarcated from the brain, with an abscess-like appearance.

Histopathological examination showed chronic granulomatous inflammation with central necrosis. Treatment was initiated with rifampin, isoniazid, pyrazinamide, and ethambutol, in addition to dexamethasone due to perilesional edema.

Central Nervous System (CNS) TB is the most severe form of systemic TB due to its high mortality rate and potential for serious neurological complications, accounting for 2 %–5 % of TB cases^1^. The most frequent manifestations are meningitis and tuberculome, meanwhile TB abscesses are present in only 10 % of these patients^2^. Symptoms typically include headache, focal neurological deficits, and seizures^3^. Therefore, even in immunocompetent patients with pulmonary TB, CNS evaluation is crucial if neurological symptoms arise.

## Ethics approval statement

We confirm that informed consent was obtained from the patient involved in this study. The patient provided written consent for the inclusion of their case details and any relevant information in the publication, in accordance with ethical and legal requirements. No artificial intelligence tools were used for image generation or text development, ensuring full compliance with legal guidelines.

## Data availability

The data that support the findings of this study are available from the corresponding author upon reasonable request.

## Funding

This study received no funding. The data were obtained from the medical records of a patient admitted to the Hospital de Clínicas de Porto Alegre.

## Conflicts of interest

The authors declare the following financial interests/personal relationships which may be considered as potential competing interests.
